# Anti-Inflammatory Activity of Triterpenes Isolated from* Protium paniculatum* Oil-Resins 

**DOI:** 10.1155/2015/293768

**Published:** 2015-12-27

**Authors:** Patrícia D. O. de Almeida, Ana Paula de A. Boleti, André Luis Rüdiger, Geane A. Lourenço, Valdir Florêncio da Veiga Junior, Emerson S. Lima

**Affiliations:** ^1^Laboratório de Atividade Biológica, Faculdade de Ciências Farmacêuticas, Universidade Federal do Amazonas (UFAM), Avenida Gen. Rodrigo Otavio, No. 6200, 69077-000 Manaus, AM, Brazil; ^2^Instituto de Ciências Exatas, Departamento de Química, Universidade Federal do Amazonas, Avenida Gen. Rodrigo Otavio, No. 6200, 69077-000 Manaus, AM, Brazil; ^3^Laboratório de Farmacologia, Departamento de Ciências Fisiológicas, Instituto de Ciências Biológicas, Universidade Federal do Amazonas, Avenida Gen. Rodrigo Otavio, No. 6200, 69077-000 Manaus, AM, Brazil

## Abstract

*Protium* is the main genus of the Burseraceae family and one of the most common genera in South America, with an important species called “breu.” Gum and oil-resins of this species are used as tonic and stimulant and for the treatment of ulcers and inflammation. The present study aims to isolate and investigate the anti-inflammatory activity of triterpene compounds isolated from oil-resin of* Protium paniculatum*. The pentacyclic triterpenes *α*,*β*-amyrin, acetylated *α*,*β*-amyrin, *α*,*β*-amyrone, and brein/maniladiol did not alter the viability of murine J774 macrophages (IC_50_ > 20 *µ*g/mL), with the exception of mixture of brein/maniladiol which showed moderate cytotoxic activity. Also it was observed that compounds at 10 *µ*g/mL inhibited more than 80% of production of NO^•^, although only *α*,*β*-amyrin was able to inhibit the production of TNF-*α* (52.03 ± 2.4%). The compounds inhibited the production of IL-6 and induced the production of IL-10 in murine J774 macrophages stimulated by LPS. *α*,*β*-Amyrone inhibited the expression of COX-2 and also inhibited the formation of paw or ear edema in rats and mice, having a quick and immediate effect. This study may provide the basis for future investigations on the therapeutic role of *α*,*β*-amyrone in treating inflammation.

## 1. Introduction

Inflammation is a defense reaction of the body, and a local response of living tissues to injury in mammalians aimed at eliminating or limiting the spread of an injurious agent [1]. The use of medicinal plants or their active components is becoming an increasingly attractive approach for treating various inflammatory disorders [2]. The origin of the anti-inflammatory properties of various phytomedicines can be explained by the presence of substances such as flavonoids, alkaloids, tannins, saponins, anthraquinones, triterpenoids, and other constituents which act as inhibitors of molecular targets and proinflammatory mediators in inflammatory responses [3].

Triterpenoids are constituents that have aroused great interest in recent years due to their pharmacological potential, with numerous therapeutic activities, such as anticancer, anti-inflammatory, antiviral, antibacterial, antifungal, antidiuretic, giardicidal, and acetylcholinesterase inhibitors [4–6]. *α*,*β*-Amyrin is a pentacyclic triterpene and constitutes the main component of the resin* Protium* sp. Furthermore, other compounds have been isolated from the resin* Protium* sp., and little is known about its anti-inflammatory properties [7].

In the last 10 years, studies have shown systemic anti-inflammatory action of *α*,*β*-amyrin associated with inhibition of the transcription factor NF-*κ*B, inhibition of COX-2, and the production of proinflammatory cytokines [7, 8]. It was recently shown that *δ*-amyrone, a constituent which is extracted and separated from of* Sedum lineare* Thunb., inhibited the ear edema in xylene-induced mouse ear edema and also decreased the level of nitric oxide (NO), prostaglandin E2 (PGE2), interleukin-6 (IL-6), and leukocyte numbers in acetic acid-induced peritonitis* in vivo* [9].

Based on evidence that* Protium* species accumulate, mainly tetracyclic and pentacyclic triterpenoids were isolated from* Protium paniculatum* mixtures of triterpenoids brein/maniladiol and *α*,*β*-amyrin [10]. Synthetic derivatives, acetylated amyrin and *α*,*β*-amyrone, were obtained from *α*,*β*-amyrin. This study aims to evaluate the anti-inflammatory activity of triterpenoids cited, considering that there are few studies in the literature showing possible biological activity.

## 2. Methods

### 2.1. Plant Material

Oleoresin of* Protium paniculatum* var.* modestum* (PPM) was collected in Ducke Forest Reserve, 26, Highway AM-010, Km 26, Manaus, AM, Brazil. The species was catalogued by the Flora Project of Ducke Reserve of the National Institute of Amazonian Research (Instituto Nacional de Pesquisas da Amazônia, INPA) and it was identified by Burseraceae taxonomists: Ph.D. Douglas C. Daly and Ph.D. José Eduardo L. S. Ribeiro. Voucher was deposited in the New York Botanical Garden (1413737) and the INPA herbarium (191303).

### 2.2. Extraction and Isolation

**Figure 1:**
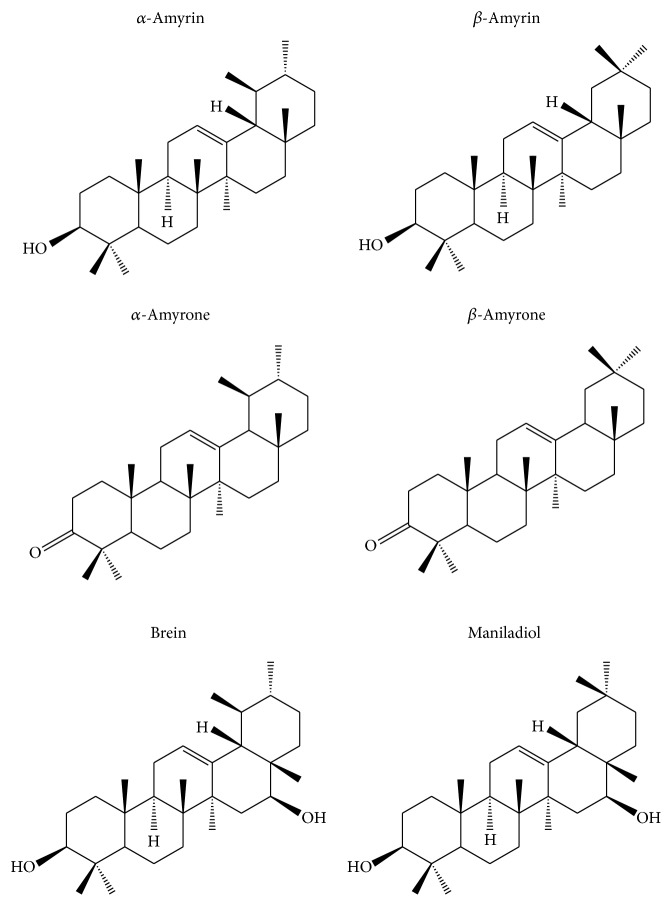
Chemical structure of the compounds isolated from* Protium* spp. resin.

Mono- and dihydroxylated triterpenes were isolated from the insoluble material which resulted from PPM oleoresin hexanic extraction (Figure 1). Samples were solubilized with ethyl acetate (1008.9 mg); this material was submitted to gravity chromatography over silica gel (mesh: 70–230, *Ø*_column_: 2.5 cm, and *m*_(SiO2)_: 40 g) using dichloromethane (DCM) and ethyl acetate with gradient polarity. Ketones and acetyl derivatives were obtained from the amyrin mixture by chemical reactions. The data relating to isolations, identification, and reactions are described in Supplementary Material available online at http://dx.doi.org/10.1155/2015/293768.

### 2.3. Cell Culture

The murine macrophage cell line J774 was kindly provided by Dr. Leda Quercia Vieira (Laboratory of Gnotobiology and Immunology, UFMG, MG, Brazil) and was cultured at 37°C in a humidified incubator with 5% CO_2_ in RPMI-1640 medium containing 10% fetal bovine serum (FBS), 50 U/mL penicillin, and 50 *μ*g/mL streptomycin (Invitrogen). Lipopolysaccharide (LPS) was prepared as a 1 mg/mL stock solution in sterile water and stored at −20°C. The triterpene compounds were added along with treatment with LPS.

### 2.4. Animals

Female Wistar rats (200 g each) and Swiss mice (25–35 g) were previously housed in standard polypropylene cages under controlled conditions of temperature (22 ± 2°C) and 12 h light/dark cycle, with free access to diet and water. Mice were allowed to adapt to laboratory for at least 1 h before testing. All experimental procedures using animals were performed following international guidelines and approved by the Institutional Animal Ethics Committee (number 002/2013 CEEA/UFAM).

### 2.5. Cell Viability Assay

The cytotoxicity of triterpenes compounds to the murine macrophage cell line J774 was determined by the Alamar Blue method as described by Nakayama and coworkers [11]. Briefly, adherent cells (5 × 10^3^ cells/well) were grown in 96-well tissue culture plates and exposed to the triterpenes: *α*,*β*-amyrin, acetylated *α*,*β*-amyrin, *α*,*β*-amyrone, and brein/maniladiol (2.5; 5; and 10 *μ*g/mL) for 24, 48, and 72 h. After incubation, the Alamar Blue solution (10 *μ*L of 0.4% Alamar Blue (resazurin) in PBS) was added and the cells were incubated for 3 h at 37°C. Fluorescence was measured (excitation at 545 nm and emission at 595 nm) and expressed as a percentage of the cells in the control after background fluorescence was subtracted. Doxorubicin (5 *μ*g/mL) was used as a positive control of cell death. The assays were performed in triplicate.

### 2.6. NO^•^ Production Assay

Nitric oxide (NO^•^) production by J774 cells was assayed by measuring the accumulation of nitrite in the culture medium using Griess reaction [12]. Briefly, after incubation of the cells (1 × 10^6^ cells/mL) with triterpenes compounds in different concentrations of 2.5; 5; and 10 *μ*g/mL, cells were incubated for 24 h with LPS (1 *μ*g/mL), at 37°C in a 5% CO_2_ incubator. Nitric oxide was measured as NO_2_^−^ in culture supernatant by reaction with Griess reagent. Absorbance of the reaction product was determined at 560 nm using a microplate reader (DTX 800, Beckman). Sodium nitrite was used as a standard to calculate nitrite.

### 2.7. Measurement of Cytokines

Macrophage cells (1 × 10^6^ cells/mL) were incubated with the triterpenes compounds in a concentration of 10 *μ*g/mL and then stimulated with 1 *μ*g/mL of LPS. The culture supernatants were collected after 24 h of LPS stimulation. The levels of cytokines in the culture media were measured by flow cytometry (BD Cytometric Bead Array, CBA, Mouse Inflammation kit) according to the manufacturer's instructions.

### 2.8. Western Blot Analysis

J774 cells were cultured in 96-well plates (1 × 10^6^ cells per well) and incubated with *α*,*β*-amyrone in concentrations of 2.5; 5; and 10 *μ*g/mL. Cells were stimulated with LPS (1 *μ*g/mL) and incubated for 24 hours. After incubation, cells were washed with phosphate buffered saline and lysed with lysis buffer (Tris-HCl [50 mM, pH 7.5]), 150 mM NaCl, 0.5% nonidet P-40, 1 mM EGTA, 1 mM MgCl_2_, 10% glycerol, and proteases inhibitors (cocktail of protease inhibitors EDTA-free, Roche; 1 mM PMSF). After 1 hour at 4°C, cell lysates were obtained by centrifugation at 10,000 g for 10 minutes. The total protein concentration in the lysates was measured by Bradford method [13], protein assay using bovine serum albumin as the standard.

Samples containing equal amounts of protein concentration were separated by 12% of sodium dodecyl sulfate-polyacrylamide gel electrophoresis and transferred to nitrocellulose membranes. Nonspecific binding was blocked with Tris-buffered saline with Tween 20 (1 M Tris-HCl [pH 7.5], 2.5 M NaCl, and 0.5% Tween 20) containing 5% nonfat milk for 2 hours at room temperature. The membranes were incubated overnight with the primary antibody [COX-2 and *β*-actin (abcam, ab52237, and ab8227, resp.)] diluted in Tris-buffered saline with Tween 20 (1 : 1.000 and 1 : 2.000, resp.) and then washed with Tris-buffered saline with Tween 20 and incubated with horseradish peroxidase-conjugated anti-immunoglobulin G antibody (goat anti-rabbit immunoglobulin G) as secondary antibody for 1 hour at room temperature. The immunoblots were visualized with a chemiluminescence detection kit, used according to the manufacturer's recommendations (SuperSignal West Pico Chemiluminescent Substrate, Prod # 34080, Thermo Scientific).

### 2.9. Carrageenan Induced Paw Edema Assay

Paw edema was induced by intraplantar injection of 100 *μ*L of 1% carrageenan into the right hind paw of rats as previously described [14]. Animal groups were treated with *α*,*β*-amyrone (10 and 5 mg/kg, v.o.) and indomethacin (10 mg/kg, v.o.) and the control animals received identical treatments with the vehicle, which was 3% Tween 80 (10 mg/kg) in saline in this study. After sixty minutes, the animals received an intraplantar injection of carrageenan. The paw volume was measured thereafter at “0 hours” and then at 1, 2, 3, 4, and 5 hours after carrageenan injection using a hydroplethysmometer (Panlab, SLU). The results are expressed as the increase in paw volume (mL) calculated by subtracting basal volume.

### 2.10. Ear Phenol-Induced Edema

Inflammation was induced in Balb C mice (*n* = 5/group) by local administration of 20 *μ*L of a solution of phenol diluted in acetone (10%) (group 1), administered after 20 *μ*L *α*,*β*-amyrone solution at concentrations of 0.6 mg, 0.3 mg, and 0.1 mg or dexamethasone of 0.1 mg dissolved in acetone. Sixty minutes after application, mice were euthanized and both ears were removed. Circular sections were removed, using a biopsy punch with a diameter of 5 mm, and weight of the inflamed ears was compared with weight of the ear against-lateral not treated with the phlogistic agent. The increase in weight caused by the irritant was measured by subtracting the weight of the untreated left ear section from that of the treated right ear sections [15].

### 2.11. Statistical Analysis

Results are expressed as the means and standard deviations of triplicate measurements. Each experiment was performed at least three times. Differences between groups were assessed by one-way analysis of variance (ANOVA) followed by the Tukey* post hoc* test. A value of *P* < 0.05 indicated significance. Western blots are representative of 3 independent experiments.

Data obtained from animal experiments were expressed as the mean ± standard error of the mean (±SEM). Statistical differences between the treated and the control groups were analyzed statistically by analysis of variance (ANOVA) followed by Dunnett's test, in the tutorial Prisma 3.0. Results with ^*∗*^ *P* < 0.05 and ^*∗∗*^ *P* < 0.01 were considered significant.

## 3. Results

Before evaluating the anti-inflammatory effects of triterpenes isolated from* Protium paniculatum* on LPS-stimulated J774 macrophages, first the cytotoxic effects were investigated. Triterpenes did not exhibit a significant reduction in viability of macrophages compared with the positive control, showing IC_50_ > 20 *μ*g/mL, except that triterpene brein/maniladiol showed IC_50_ = 16.02 *μ*g/mL after 24 hours of treatment (Table 1).

**Figure 2:**
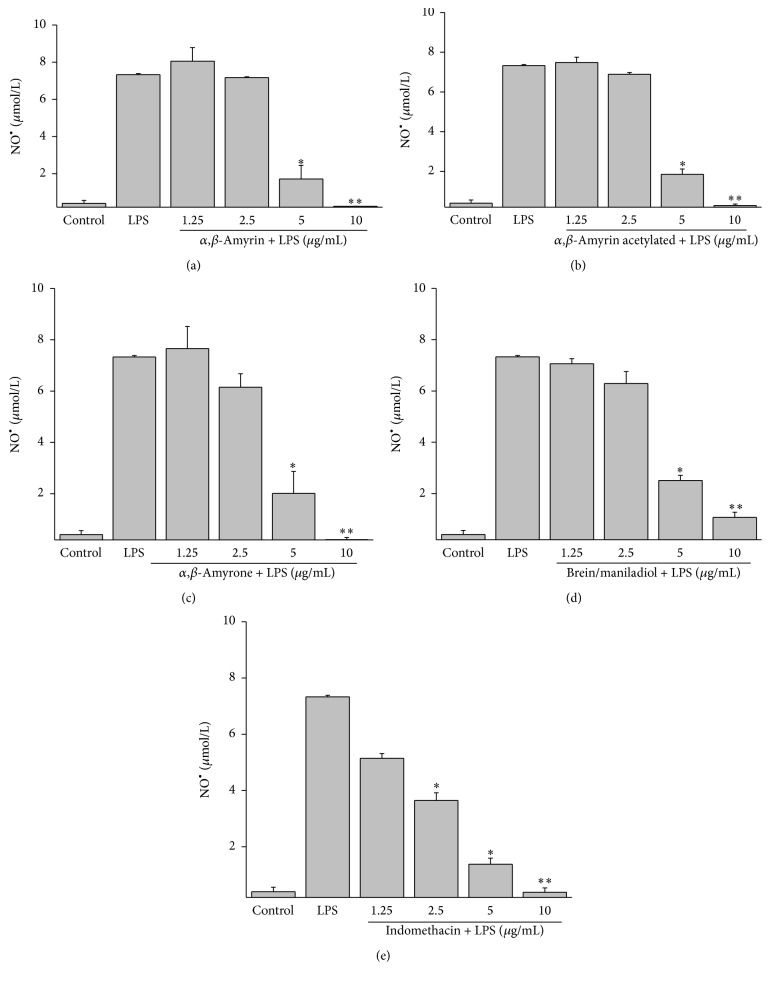
Effect of the isolated triterpenes on NO^•^ production in LPS-stimulated J774 cells. (a) *α*,*β*-amyrin (b) acetylated *α*,*β*-amyrin (c) *α*,*β*-amyrone (d) brein/maniladiol, and (e) indomethacin. Production of NO^•^ was assayed in culture supernatants of macrophages stimulated with LPS (1 *μ*g/mL) for 24 h in the presence of the four compounds (1.25–10 *μ*g/mL). The nitrite values are the mean ± SD from three independent experiments. Significance was determined using Student's-*t*-test (^*∗*^ *P* < 0.05; ^*∗∗*^ *P* < 0.01 compared to LPS).

Because NO^•^ is known to be a proinflammatory mediator in inflammatory disorders [16], we investigated whether triterpenes inhibit NO^•^ production in LPS-induced J774 cells. We measured the accumulation of nitrite in the culture media and found that triterpenes concentration-dependently inhibited nitrite levels in the conditioned media of LPS-induced cells.  Figure 2 shows the inhibitory effect of triterpenes, *α*,*β*-amyrin, acetylated *α*,*β*-amyrin, *α*,*β*-amyrone, and brein/maniladiol, on NO^•^ production at concentration of 1.25–10 *μ*g/mL. The triterpenes inhibited the production of NO^•^ at 98.34 ± 0.9%; 96.05 ± 0.8%; 99.86 ± 1.1%; and 75.43 ± 2.8%, at 10 *μ*g/mL, respectively, and showed IC_50_ at 4.96 ± 0.2; 5.04 ± 0.12; 4.61 ± 0.08; and 6.49 ± 0.02 *μ*g/mL at 10 *μ*g/mL, respectively (Figures 2(a), 2(b), 2(c), and 2(d)). Indomethacin was used with the positive control of anti-inflammatory effect showing an inhibition of 86.31 ± 1.2% in NO^•^ production at 10 *μ*g/mL (Figure 2(e)).

**Figure 3:**
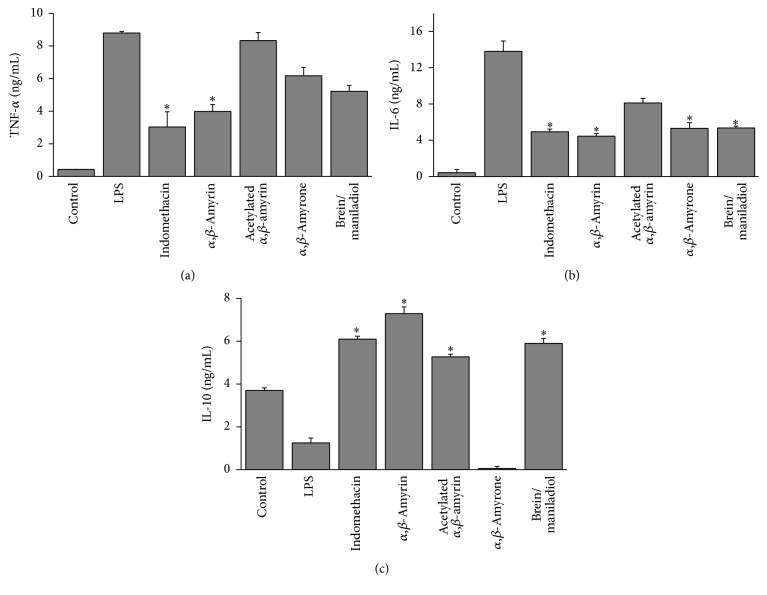
Effect of the isolated triterpenes on cytokine production in LPS-stimulated J774 cells. (a) TNF-*α*, (b) IL-6, and (c) IL-10. Indomethacin (10 *μ*g/mL) was used as a standard. The production of cytokines was assayed in the culture supernatants of macrophages stimulated with LPS (1 *μ*g/mL) for 24 h in the presence of the four compounds (10 *μ*g/mL). Each value was the mean ± SD from three independent experiments. The significance was determined using Student's-*t*-test (^*∗*^ *P* < 0.05 compared to LPS).

LPS induce production of proinflammatory cytokines such as the tumor necrosis factor-*α* (TNF-*α*) and IL-1 and IL-6 in cells. As shown in Figure 3, among the triterpenes evaluated, only the *α*,*β*-amyrin led to a significant decrease in TNF-*α* levels (52.03 ± 2.4%) at a concentration of 10 *μ*g/mL (Figure 3(a)). However, the other triterpenes, with exception of acetylated *α*,*β*-amyrin, inhibited the production of IL-6.  Figure 3(b) shows that *α*,*β*-amyrin, *α*,*β*-amyrone, brein/maniladiol, and indomethacin at a concentration of 10 *μ*g/mL inhibited the IL-6 levels at 67.81 ± 2.8%; 61.43 ± 3.2%; 61.27 ± 5.1%; and 64.24 ± 2.8%, respectively. Furthermore only *α*,*β*-amyrone showed an inhibition in IL-10 level; an anti-inflammatory cytokine is secreted under different conditions of immune activation by a variety of cell types, including T cells, B cells, and monocytes/macrophages (Figure 3(c)).

**Figure 4:**
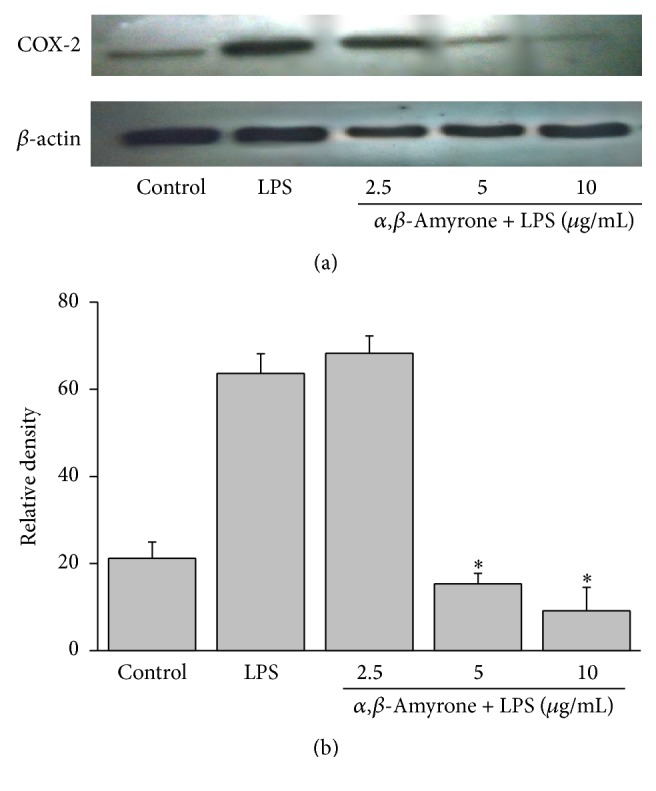
Effect of the triterpene *α*,*β*-amyrone isolated from* Protium* ssp. on COX-2 expression in LPS-stimulated J774 cells. (a) J774 cells were pretreated with concentrations of 2.5, 5, and 10 *μ*g/mL of *α*,*β*-amyrone and LPS (1 *μ*g/mL) for 24 h. The cells were lysed, and the lysates were analyzed by immunoblotting with an anti-COX-2 antibody. The blot was stripped and reprobed with an anti-actin antibody to confirm equal loading. (b) Relative density of COX-2 protein was performed using the ImageJ Software. The significance was determined using ANOVA (^*∗*^ *P* < 0.05 compared to LPS).

Due to acetylation, *α*,*β*-amyrin did increase inhibition of NO^•^ and TNF-*α*, and despite the fact that brein/maniladiol showed potential anti-inflammatory activity, it exhibited moderate cytotoxicity activity in J774 murine macrophage cells. Moreover, it is a mixture that needs to be further isolated and characterized. For these reasons we evaluated only triterpene *α*,*β*-amyrone. Thus, the protein expression levels of the COX-2 in LPS-challenged cells with and without the treatment of *α*,*β*-amyrone were evaluated by western blotting (Figures 4(a) and 4(b)). Treatment with *α*,*β*-amyrone showed inhibited COX-2 expression in a concentration-dependent manner, reduced by approximately 90%, at concentrations of 5 or 10 *μ*g/mL.

**Figure 5:**
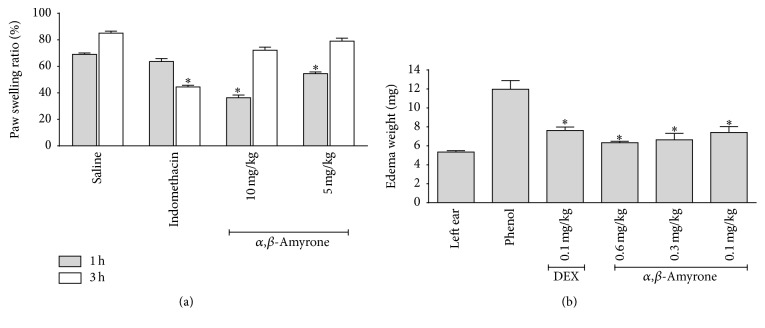
Effect of triterpene *α*,*β*-amyrone on rats paw edema induced by 1% of solution of carrageenan into the intraplantar surface of right hind paw and the effects of *α*,*β*-amyrone on mice ear edema induced by a phenol model. (a) *α*,*β*-amyrone was administered at concentrations of 5 and 10 mg/kg and the edema was measured at the indicated times. The effect of saline injected in the control group is also shown. (b) *α*,*β*-amyrone was administered at concentrations of 0.6, 0.3, and 0.1 mg/kg and dexamethasone of 0.1 mg/kg on mice ear edema induced by a phenol model. Data are expressed as mean ± standard error of five animals per group. The significance was determined using ANOVA and Dunnett's test (^*∗*^ *P* < 0.05 compared with control group).

Figure 5 shows that oral administration of *α*,*β*-amyrone (5 and 10 mg/kg, *n* = 5) induced dose-dependent rat paw edema compared with animals receiving only saline. The edema was of rapid onset and relatively short duration (36.3 ± 1.6% and 54.5 ± 1.1%, resp.), after 1 hour of treatment compared to the edema after 3 hours (72.2 ± 1.3% and 79.1 ± 0.4%, resp.). In phenol-induced ear edema in a murine model, it was found that the triterpene *α*,*β*-amyrone exhibited significant inhibition in ear edema formation in a dose-related manner. It caused 47% inhibition at the dose of 0.6 mg/kg body weight, respectively, compared with the standard drug dexamethasone where the inhibition was 36% at the dose of 0.1 mg/kg body weight Figure 5(b).

## 4. Discussion

The use of natural products, especially those derived from medicinal plants, is a traditional form of providing relief from illness. Over the years, natural products have contributed enormously to the development of important therapeutic drugs used currently in modern medicine [17, 18]. Recent studies have shown that the resin of* Protium* sp. displays marked anti-inflammatory activity, in different models of inflammation, with hepatoprotective potential, topical anti-inflammatory action, pancreatic injury, and colitis [5, 6, 18, 19].

Our study demonstrated for the first time the cytotoxic and anti-inflammatory effects of natural triterpenes *α*,*β*-amyrin, brein/maniladiol, and synthetic triterpenes acetylated *α*,*β*-amyrin and *α*,*β*-amyrone on LPS-stimulated J774 macrophages. The triterpenes did not exhibit a significant reduction in macrophage viability compared with the positive control, showing IC_50_ > 20 *μ*g/mL, except triterpene brein/maniladiol which showed IC_50_ = 16.02 *μ*g/mL in 24 hours of treatment. Similar results were observed by Siani et al. [20] who verified that essential oil obtained by steam distillation (leaves and resin) from* Protium* species at 100 *μ*g/mL inhibited the proliferation of different cell lines, with 76–89% inhibition of J774 cells after 72 h of treatment. As noted, brein/maniladiol showed cytotoxic effects on J774 macrophages. Likewise, Ukiya et al. [7] showed that maniladiol isolated from the nonsaponifiable lipid fraction of the edible flower extract of* Chrysanthemum morifolium* exhibited moderate cytotoxicity in kidney cancer cell lines and accentuated activity in breast cancer.

NO^•^ plays an important role in various inflammatory conditions where it is produced by the inducible form of nitric oxide synthase (iNOS) from the amino acid L-arginine [21, 22]. NO^•^ in tissue is susceptible to manipulation by proinflammatory cytokines [23]. NO^•^ has important immune, cardiovascular, and neurological second messenger functions implicated in sepsis, cancer, and inflammation. A variety of stimuli, such as with LPS, TNF-*α*, and IFN-*γ*, can result in the production of a massive amount of NO^•^ by the activated macrophages which can participate in the pathological processes in several acute and chronic inflammatory disorders [24]. Our results suggest that triterpenes from* P. paniculatum* have dose-dependent anti-inflammatory activities related to their inhibition of NO^•^ in macrophages without affecting the viability of these cells.

Our results were better than those obtained by Siani et al. [20] who demonstrated that essential oil obtained from leaves and resin from* Protium* species at 100 *μ*g/well, changed the NO^•^ production from stimulated mouse macrophage after 24 hours of pleurisy induction, in which the resin of* P. heptaphyllum* inhibited 74% and* P. strumosum* inhibited 46% of the NO^•^ production. In contrast, the triterpenes isolated from* Protium paniculatum*, *α*,*β*-amyrin, acetylated *α*,*β*-amyrin, *α*,*β*-amyrone, and brein/maniladiol, at a concentration of 10 *μ*g/mL inhibited the production of NO^•^ at 98.34 ± 0.9%; 96.05 ± 0.8%; 99.86 ± 1.1%; and 75.43 ± 2.8%, respectively.

Furthermore, the media of IC_50_ of triterpenes were similar with the media observed by Niu et al. [25] who evaluated their potential to inhibit the NO^•^ production induced by LPS stimulation in RAW 264.7 macrophages of one new olean-13(18)-ene-3,12,19-trione, and two known oleanene triterpenes *δ*-amyrone and *δ*-amyrin acetate isolated from a petroleum ether fraction from an alcohol extract of the whole plant of* Sedum linear* Thunb., which exhibited values of IC_50_ at 9.91 *μ*M, 12.24 *μ*M, and 43.34 *μ*M, respectively.

Monocytes and macrophages are key players in inflammatory responses and are also major sources of proinflammatory cytokines and enzymes including tumor necrosis factor-*α* (TNF-*α*), interleukins (ILs), cyclooxygenase (COX), and nitric oxide synthase (NOS) [24, 26]. These genes of proinflammatory mediators are strongly induced during inflammation and are responsible for its initiation and persistence. TNF-*α* are cytokines that act as signaling molecules for immune cells and coordinate the inflammatory response [24]. In this study, among the triterpenes tested, only *α*,*β*-amyrin inhibited TNF-*α* production. This result corroborates with several studies of inhibitory effects of *α*,*β*-amyrin on TNF-*α* production in different models of inflammation [5, 8, 18, 27].

Interleukin-6 (IL-6) is one of the earliest and most important proinflammatory cytokines produced in response to inflammatory stimuli [28]. The presence of IL-6 in tissues is not an unusual occurrence, but its production can lead to uncontrolled exposure and subsequent chronic inflammation, and they are strongly associated with many types of cancer [29]. As our continuing research on anti-inflammatory agents, a number of plant extracts and natural products have been discovered to suppress the secretion of IL-6 in LPS-stimulated macrophages* in vitro* [28, 30]. Interestingly, all the triterpenes tested exerted inhibitory effects on IL-6 production at 10 *μ*g/mL, except acetylated *α*,*β*-amyrin. Similar results were demonstrated by Lee et al. [31] who isolated seven flavonoids from the methanol extracts of* Psoralea corylifolia* (bakuchiol, bavachinin, neobavaisoflavone, corylifol A, corylin, isobavachalcone, and bavachin) and found that these compounds were able of inhibit IL-6 production by action of STAT3 promoter activity in Hep3B cells. These compounds also inhibited STAT3 phosphorylation induced by IL-6 in Hep3B cells.

Interleukin-10 (IL-10) is produced by activated macrophages and T cells and plays an important role in anti-inflammatory responses, including the inhibition of cytokine production (tumor necrosis factor-*α*, IL-6, and IL-12) in macrophages induced by lipopolysaccharide [31]. This study verified that triterpenes, except *α*,*β*-amyrone, showed induced IL-10 production. So, the decline of TNF-*α* accumulation in our study was consistent with findings in several studies demonstrating that IL-10 can suppress TNF-*α* production in human monocytes and macrophages or even cause diminished levels of TNF-*α* and IL-6 [32, 33].

Similar results were observed by Zdzisiñska et al. [34] who evaluated the immunomodulatory properties of triterpene betulin and its oxidized form, betulinic acid, as agents inducing cytokines examining human whole blood stimulated by mitogens (PHA). It was observed that triterpene betulin induced TNF-*α* production in a dose-dependent manner but did not induce the production of IL-10 and IFN-*γ*; these results suggest that secretion of IFN-*γ*, IL-10, and TNF-*α* can be regulated by different mechanisms or various types of leukocytes in whole blood differ in their sensitivity to betulin, unlike, betulinic acid, which did not influence the TNF-*α* production but inhibited the production of IFN-*γ* and increased production of IL-10.

Cyclooxygenases are inducible enzymes that catalyze the production of prostaglandins, which contribute to the inflammatory process and tissue damage. It has been reported that COX-2 can also be activated by high concentrations of nitric oxide, contributing towards more intense inflammatory responses as seen in many chronic inflammatory disorders [22]. In the current study, we verified only Cox-2 expression of triterpene *α*,*β*-amyrone that was able to inhibit COX-2 in a concentration-dependent manner. Several natural products of plant origin have been shown to transmit their anti-inflammatory activities through suppression of COX-2; however, suppression of nitric oxide production is critical for this [22, 35]. In previous studies the ability of triterpene *α*,*β*-amyrin to inhibit COX-2 expression using a different model of inflammation is shown, as in the case of topic inflammation in rats and a colitis model [2, 18]. In accordance with the prepreliminary results *α*,*β*-amyrone was able to inhibit the production of NO^•^, IL-6, and COX-2 expression, suggesting that the mechanism by which *α*,*β*-amyrone exerts its anti-inflammatory activity is the same mechanism by which *α*,*β*-amyrin acts, that is, by inhibiting the nuclear factor-kappa B (NF-*κ*B).

The model of paw edema induced by carrageenan is an appropriate test and widely used for evaluating anti-inflammatory activity of different compounds [1]. Carrageenan induced hind paw edema is the standard experimental model of acute inflammation. Carrageenan is the phlogistic agent of choice for testing anti-inflammatory drugs as it is not known to be antigenic and is devoid of apparent systemic effects [36]. The present study of anti-inflammatory activity of *α*,*β*-amyrone against carrageenan induced paw edema shows that triterpenes have a significant effect on inflammation and markedly reduced the swelling at 10 *μ*g/mL after 3 hours of treatment.

*α*,*β*-Amyrone showed the activity at concentrations of 10 and 5 mg/kg and showed a different effect of indomethacin, whereas *α*,*β*-amyrone showed a maximum effect in the first hour after administration of carrageenan with a decrease with respect to time, and indomethacin at a dose of 10 mg/kg showed increased activity after three hours of induction of inflammation, when the carrageenan starts to show its greatest inflammatory effect, decreasing effect in five hours, which indicates an effect faster than *α*,*β*-amyrone.

In the present study, the significant anti-inflammatory effect of topical application of triterpene *α*,*β*-amyrone in phenol-induced mouse ear edema was shown for the first time. Phenol is an irritant agent for stimulating contact dermatitis in mice [37, 38]. Skin keratinocyte membranes are ruptured upon direct contact with phenol, resulting in protein kinase C mediating release of inflammatory mediators such as IL-1*α*, TNF-*α*, and IL-8 [38–40]. The topical anti-inflammatory activity of *α*,*β*-amyrone was demonstrated by results showing that *α*,*β*-amyrone dose-dependently attenuated the phenol-induced ear edema with an effect as potent as dexamethasone and showing the property of this substance to penetrate the skin and exert its activity in deeper layers which could be indicator of its potential use in pharmaceutical formulations with anti-inflammatory properties. In addition, the anti-inflammatory activity of *α*,*β*-amyrone needs the additional studies which will provide clinical evidences in context of specific inflammatory inductions and/or microbial infection activity.

## 5. Conclusion

The triterpenes *α*,*β*-amyrin, acetylated *α*,*β*-amyrin, *α*,*β*-amyrone, and brein/maniladiol are capable of modulating an immune response. In particular, the triterpene *α*,*β*-amyrone showed no cytotoxic potential in J774 macrophages and exerted immunomodulatory activity at low concentrations, characterized by its inhibitory effects on the production of proinflammatory mediators such as NO^•^, IL-6, and COX-2 expression and inducing the production of anti-inflammatory cytokine IL-10, and reduced paw edema induced by carrageenan in rats, as well as reducing ear edema in mice.

## Supplementary Material

The data relating to isolations, identification, RNM data and reactions are described in section of supplementary material.

## Figures and Tables

**Table 1:** Cell viability of J774 cells treated with 5, 10, and 20 *μ*g/mL of isolated triterpenes for 24, 48, and 72 hours.

	24 hours			48 hours			72 hours		
Concentration (*µ*g/mL)	20	10	5	20	10	5	20	10	5
	Mean ± SE	Mean ± SE	Mean ± SE	Mean ± SE	Mean ± SE	Mean ± SE	Mean ± SE	Mean ± SE	Mean ± SE
*α*,*β*-Amyrin	57.3 ± 1.9	86.4 ± 0.8	105.8 ± 2.3	36.4 ± 3.1	88,5 ± 2.8	110.0 ± 2.5	37.1 ± 3.5	88.7 ± 1.2	97.2 ± 1.8
Acetylated *α*,*β*-amyrin	87.1 ± 0.5	152.9 ± 0.7	124.4 ± 1.9	32.1 ± 2.4	99.5 ± 3.6	125.0 ± 1.7	47.7 ± 1.9	98.0 ± 2.9	103.5 ± 2.7
*α*,*β*-Amyrone	119.4 ± 0.5	153.8 ± 1.8	144.0 ± 0.78	81.9 ± 2.9	126.7 ± 0.7	133.5 ± 1.5	65.3 ± 1.7	102.0 ± 0.7	105.2 ± 3.5
Brein/maniladiol	40.6 ± 1.1	82.4 ± 1.9	97.2 ± 3.5	13.3 ± 2.0	47.6 ± 2.9	90.0 ± 1.2	5.4 ± 0.6	23.8 ± 3.1	80.2 ± 1.6
Indomethacin	100.1 ± 3.2	123.6 ± 2.9	117.5 ± 3.7	50.5 ± 2.8	100.8 ± 1.3	105.9 ± 0.8	57.9 ± 1.1	102.3 ± 1.7	103.8 ± 2.9
Doxorubicin	26.0 ± 0.4	27.3 ± 1.2	25.7 ± 2.6	9.3 ± 1.4	10.0 ± 0.1	10.1 ± 0.2	3.8 ± 0.7	3.9 ± 0.07	3.9 ± 0.1
DMSO	70.8 ± 0.7	108.6 ± 0.6	113.3 ± 0.6	38.9 ± 0.2	98.9 ± 2.7	109.5 ± 2.9	33.4 ± 2.3	98.6 ± 1.1	99.0 ± 1.3
Medium	106.1 ± 4.0	100.0 ± 9.7	101.30 ± 1.5	106.7 ± 1.8	101.9 ± 11.0	103.4 ± 2.1	99.9 ± 4.8	98.9 ± 3.4	95.4 ± 4.2

*Notes*. Data are presented as % mean ± standard error (*n* = 3). SE: standard error; DMSO: dimethyl sulfoxide.

## Abbreviations

COX_2_: Cyclooxygenase 2
Dexa: Dexamethasone
DMSO: Dimethyl sulfoxide
EDTA: Ethylenediaminetetraacetic acid
EGTA: Ethylene glycol tetraacetic acid
IL6: Interleukin-6
IL10: Interleukin-10
LPS: Lipopolysaccharide
MgCl_2_: Magnesium chloride
NaCl: Sodium chloride
NF-*κ*B: Nuclear factor kappa B
NO: Nitric oxide
PBS: Phosphate buffered saline
PMSF: Phenylmethanesulfonyl fluoride
TNF*α*: Tumor necrosis factor-*α*.

## References

[1] Patgiri B., Umretia B. L., Vaishnav P. U., Prajapati P. K., Shukla V. J., Ravishankar B. (2014). Anti-inflammatory activity of *Guduchi Ghana* (aqueous extract of *Tinospora cordifolia* Miers.). *AYU*.

[2] Vitor C. E., Figueiredo C. P., Hara D. B., Bento A. F., Mazzuco T. L., Calixto J. B. (2009). Therapeutic action and underlying mechanisms of a combination of two pentacyclic triterpenes, *α* and *β*-amyrin, in a mouse model of colitis. *British Journal of Pharmacology*.

[3] Soumaya K.-J., Dhekra M., Fadwa C. (2013). Pharmacological, antioxidant, genotoxic studies and modulation of rat splenocyte functions by *Cyperus rotundus* extracts. *BMC Complementary and Alternative Medicine*.

[4] Bandeira P. N., Pessoa O. D. L., Trevisan M. T. S., Lemos T. L. G. (2002). Metabólitos secundários de *Protium heptaphyllum* March. *Química Nova*.

[5] Melo C. M., Carvalho K. M. M. B., de Sousa Neves J. C. (2010). *α*,*β*-amyrin, a natural triterpenoid ameliorates L-arginine-induced acute pancreatitis in rats. *World Journal of Gastroenterology*.

[6] Matos I., Bento A. F., Marcon R., Claudino R. F., Calixto J. B. (2013). Preventive and therapeutic oral administration of the pentacyclic triterpene *α*,*β*-amyrin ameliorates dextran sulfate sodium-induced colitis in mice: the relevance of cannabinoid system. *Molecular Immunology*.

[7] Ukiya M., Akihisa T., Tokuda H. (2002). Constituents of compositae plants. III. Anti-tumor promoting effects and cytotoxic activity against human cancer cell lines of triterpene diols and triols from edible chrysanthemum flowers. *Cancer Letters*.

[8] da Silva K. A. B. S., Paszcuk A. F., Passos G. F. (2011). Activation of cannabinoid receptors by the pentacyclic triterpene *α*,*β*-amyrin inhibits inflammatory and neuropathic persistent pain in mice. *Pain*.

[9] Niu X., Yao H., Li W. (2014). *δ*-Amyrone, a specific inhibitor of cyclooxygenase-2, exhibits anti-inflammatory effects in vitro and in vivo of mice. *International Immunopharmacology*.

[10] Rüdiger A. L., Veiga-Junior V. F. (2013). Chemodiversity of ursane- and oleanane-type triterpenes in amazonian burseraceae oleoresins. *Chemistry & Biodiversity*.

[11] Nakayama G. R., Caton M. C., Nova M. P., Parandoosh Z. (1997). Assessment of the Alamar Blue assay for cellular growth and viability in vitro. *Journal of Immunological Methods*.

[12] Green L. C., Wagner D. A., Glogowski J., Skipper P. L., Wishnok J. S., Tannenbaum S. R. (1982). Analysis of nitrate, nitrite, and [^15^N]nitrate in biological fluids. *Analytical Biochemistry*.

[13] Bradford M. M. (1976). A rapid and sensitive method for the quantitation of microgram quantities of protein utilizing the principle of protein-dye binding. *Analytical Biochemistry*.

[14] Winter C. A., Risley E. A., Nuss G. M. (1962). Carrageenin-induced edema in hind paw of the rat as an assay for antiiflammatory drugs. *Proceedings of the Society for Experimental Biology and Medicine*.

[15] Kondo S., Fujisawa S. H., Shivji G. M. (1995). Interleukin-1 receptor antagonist suppresses contact hypersensitivity. *Journal of Investigative Dermatology*.

[16] Nathan C. (1992). Nitric oxide as a secretory product of mammalian cells. *The FASEB Journal*.

[17] Surh Y.-J., Kundu J. K., Na H.-K., Lee J.-S. (2005). Redox-sensitive transcription factors as prime targets for chemoprevention with anti-inflammatory and antioxidative phytochemicals. *Journal of Nutrition*.

[18] Medeiros R., Otuki M. F., Avellar M. C. W., Calixto J. B. (2007). Mechanisms underlying the inhibitory actions of the pentacyclic triterpene *α*-amyrin in the mouse skin inflammation induced by phorbol ester 12-*O*-tetradecanoylphorbol-13-acetate. *European Journal of Pharmacology*.

[19] Oliveira F. A., Chaves M. H., Almeida F. R. C. (2005). Protective effect of *α*- and *β*-amyrin, a triterpene mixture from *Protium heptaphyllum* (Aubl.) March. trunk wood resin, against acetaminophen-induced liver injury in mice. *Journal of Ethnopharmacology*.

[20] Siani A. C., Ramos M. F. S., Menezes-De-Lima O. (1999). Evaluation of anti-inflammatory-related activity of essential oils from the leaves and resin of species of *Protium*. *Journal of Ethnopharmacology*.

[21] Kojda G., Harrison D. (1999). Interactions between NO and reactive oxygen species: pathophysiological importance in atherosclerosis, hypertension, diabetes and heart failure. *Cardiovascular Research*.

[22] Abdelwahab S. I., Koko W. S., Taha M. M. E. (2012). In vitro and in vivo anti-inflammatory activities of columbin through the inhibition of cycloxygenase-2 and nitric oxide but not the suppression of NF-*κ*B translocation. *European Journal of Pharmacology*.

[23] Wimalawansa S. J. (2008). Nitric oxide: new evidence for novel therapeutic indications. *Expert Opinion on Pharmacotherapy*.

[24] Verma N., Tripathi S. K., Sahu D., Das H. R., Das R. H. (2010). Evaluation of inhibitory activities of plant extracts on production of LPS-stimulated pro-inflammatory mediators in J774 murine macrophages. *Molecular and Cellular Biochemistry*.

[25] Niu X.-F., Liu X., Pan L., Qi L. (2011). Oleanene triterpenes from *Sedum lineare* Thunb. *Fitoterapia*.

[26] Bonizzi G., Karin M. (2004). The two NF-*κ*B activation pathways and their role in innate and adaptive immunity. *Trends in Immunology*.

[27] Pinto S. A. H., Pinto L. M. S., Cunha G. M. A., Chaves M. H., Santos F. A., Rao V. S. (2008). Anti-inflammatory effect of *α*, *β*-Amyrin, a pentacyclic triterpene from *Protium heptaphyllum* in rat model of acute periodontitis. *Inflammopharmacology*.

[28] Ko H. N., Oh T.-H., Baik J. S., Hyun C.-G., Kim S. S., Lee N. H. (2013). Anti-inflammatory activities for the extracts and carpinontriols from branches of *Carpinus turczaninowii*. *International Journal of Pharmacology*.

[29] Hodge D. R., Hurt E. M., Farrar W. L. (2005). The role of IL-6 and STAT3 in inflammation and cancer. *European Journal of Cancer*.

[30] Yang S.-K., Wang Y.-C., Chao C.-C., Chuang Y.-J., Lan C.-Y., Chen B.-S. (2010). Dynamic cross-talk analysis among TNF-R, TLR-4 and IL-1R signalings in TNF*α*-induced inflammatory responses. *BMC Medical Genomics*.

[31] Lee S. W., Yun B. R., Kim M. H. (2012). Phenolic compounds isolated from *Psoralea corylifolia* inhibit IL-6-induced STAT3 activation. *Planta Medica*.

[32] Kuwata H., Watanabe Y., Miyoshi H. (2003). IL-10-inducible Bcl-3 negatively regulates LPS-induced TNF-*α* production in macrophages. *Blood*.

[33] Chanput W., Mes J., Vreeburg R. A. M., Savelkoul H. F. J., Wichers H. J. (2010). Transcription profiles of LPS-stimulated THP-1 monocytes and macrophages: a tool to study inflammation modulating effects of food-derived compounds. *Food & Function*.

[34] Zdzisiñska B., Rzeski W., Paduch R. (2003). Differential effect of betulin and betulinic acid on cytokine production in human whole blood cell cultures. *Polish Journal of Pharmacology*.

[35] Tian J., Kim S. F., Hester L., Snyder S. H. (2008). S-nitrosylation/activation of COX-2 mediates NMDA neurotoxicity. *Proceedings of the National Academy of Sciences of the United States of America*.

[36] Ganguly A., Mahmud Z. A., Uddin M. M. N., Rahman S. M. A. (2013). In-vivo anti-inflammatory and anti-pyretic activities of *Manilkara zapota* leaves in albino Wistar rats. *Asian Pacific Journal of Tropical Disease*.

[37] Lim H., Park H., Kim H. P. (2004). Inhibition of contact dermatitis in animal models and suppression of proinflammatory gene expression by topically applied flavonoid, wogonin. *Archives of Pharmacal Research*.

[38] Mo J., Panichayupakaranant P., Kaewnopparat N., Songkro S., Reanmongkol W. (2014). Topical anti-inflammatory potential of standardized pomegranate rind extract and ellagic acid in contact dermatitis. *Phytotherapy Research*.

[39] Wilmer J. L., Burleson F. G., Kayama F., Kanno J., Luster M. I. (1994). Cytokine induction in human epidermal keratinocytes exposed to contact irritants and its relation to chemical-induced inflammation in mouse skin. *Journal of Investigative Dermatology*.

[40] Saraiva R. A., Araruna M. K. A., Oliveira R. C. (2011). Topical anti-inflammatory effect of *Caryocar coriaceum* Wittm. (Caryocaraceae) fruit pulp fixed oil on mice ear edema induced by different irritant agents. *Journal of Ethnopharmacology*.

