# Supplementary material for: Anti-Inflammatory Activity of Triterpenes Isolated from Protium paniculatum Oil-Resins
Source: Evid Based Complement Alternat Med. 2015 Dec 27;2015:293768. doi: 10.1155/2015/293768 (PMC4806667; doi:10.1155/2015/293768)
Supplement: Supplementary file 1 — The data relating to isolations, identification, RNM data and reactions are described in section of supplementary material. [file 293768.f1.doc]

**Supplementary material**

**General Experimental Procedures.** The NMR spectra were recorded on Varian INOVA 500 NMR spectrometers. The Massa Spectra were obtained used gas chromatograph equipped with Massa Spectra detector QP-2010 for Shimadzu, used capillary column VF-1MS (15m x 0.25mm id x 0.25μm film thickness). The mass spectra were obtained used 70eV.

**Plant Material.** Oleoresin of *Protium paniculatum* var. *modestum* (PPM) was collected in Ducke Forestry Reserve, 26, Highway AM-010, Km 26, Manaus – AM, Brazil, the species was catalogued by the Flora Project of Ducke Reserve of the National Institute of Amazonian Research (Instituto Nacional de Pesquisas da Amazônia – INPA) and your identification realized by the Burseraceae Taxonomists Ph.D. Douglas C. Daly and Ph.D. José Eduardo L.S Ribeiro. The exicate were depositaded in New York Botanical Garden (1413737) and the INPA herbarium (191303).

**Extraction and Isolation.** The resin was washed with hexane in order to separate (by solubilization) the apolar light oil (composed by monoterpenes and sesquiterpenes) and a great part of the monohidroxilated triterpenes (amyrins) from di-hidroxylated and carbonilic triterpenes. The residue was solubilized in ethyl acetate, in order to obtain the so-called ethyl acetate extract of oleoresin of PPM. The ethyl acetate extract of PPM (1008.9 mg) was submitted to gravity chromatography over silica gel (mesh:70-230, Ø_column_: 2.5 cm, m_(SiO2)_: 40 g) using dichloromethane (DCM) and ethyl acetate with gradient polarity. The second fraction [PPM-1] (obtained on DCM:Ethyl Acetate 2%), was crystallized with ethanol provided the whitish crystals of α- amyrin (1) and β-amyrin (2) mixture (504.6 mg), part of the amyrins not solubilized with hexane. The third fraction [PPM-2] (obtained on DCM: Ethyl acetate 5%), was crystallized with ethanol to provide the whitish crystals of brein (3) and maniladiol (4) mixture (68.4 mg). The fifth fraction [PPM-3] (obtained on DCM: Ethyl acetate 15%), was crystallized with ethyl acetate: methanol (6:1) provided the transparent hexagonal crystals of 3α-hydroxytirucalla-7,24-diene-21-oic acid (5) and 3α-hydroxytirucalla-8,24-diene-21-oic acid (6) in a mixture (47.2 mg). The gravity chromatography was repeated twice to obtain larger quantities of fractions.

The α-amyrone (**1a**) and β-amyrone (**2a**) mixture was obtained by oxidation of **1** and **2** mixture (500 mg) using pyridinum chlorochromate (336.6 mg) in DCM, before the product was then extracted and filtered over silica gel using hexane as the eluent, and dried to yield 432.6 mg of PPM-1a (containing the **1a** and **2a** mixture).^1,2^ The 3-acethyl-*O*-α-amyrin (1b) and 3-acethyl-*O*-β-amyrin (1c) mixture was obtained by acetylation of the fraction PPM-1 (100 mg) with 10 mL of pyridine and 10 mL of acetic anhydride. After evaluation by TLC, the reaction was terminated by adding drops of H_2_SO_4_ to the formation of white precipitates. The solution was washed with chloroform and HCl repeatedly, always discarding the aqueous fraction. At the end, the chloroform fraction was washed with distilled water, and the organic fraction was concentrated and dried to yeld PPM-1b (containing the **1a** and **2a** mixture). The fractions were analyzed by GC-MS and multiple NMR (^1^H, ^13^C and DEPT). The identification parameters were compared with recent publication of literature (3).

**Fraction PPM-1:** Isolated as an 2:1 mixture of α-amyrin **(1)** and β-amyrin **(2);** ^1^H NMR **(1)** (CDCl_3_, 500 MHz): δ 3.23 (dd, J=11.0, 5.2 Hz, H-3), 5.14 (t, *J*=3.7 Hz, H-12); ^1^H NMR **(2)** (CDCl_3_, 500 MHz): δ 3.23 (dd, J=11.0, 5.2 Hz, H-3), 5.19 (t, *J*=3.7 Hz, H-12); ^13^C NMR **(1)** (CDCl_3_, 125 MHz) δ 38.8 (CH_2_, C-1), 27.3 (CH_2_, C-2), 79.1 (CHOH, C-3), 38.8 (C, C-4), 55.2 (CH, C-5), 18.4 (CH_2_, C-6), 33.0 (CH_2_, C-7), 40.0 (C, C-8), 47.7 (CH, C-9), 36.9 (C, C-10), 23.4 (CH_2_, C-11), 124.4 (HC=, C-12), 139.6 (C=, C-13), 42.1 (C, C-14), 28.1 (CH_2_, C-15), 26.6 (CH_2_, C-16), 33.8 (C, C-17), 59.1 (CH, C-18), 39.6 (CH, C-19), 39.7 (CH, C-20), 31.3 (CH_2_, C-21), 41.5 (CH_2_, C-22), 28.1 (CH_3_, C-23), 15.6 (CH_3_, C-24), 15.7 (CH_3_, C-25), 16.9 (CH_3_, C-26), 23.3 (CH_3_, C-27), 28.8 (CH_3_, C-28), 17.5 (CH_3_, C-29), 21.4 (CH_3_, C-30); ^13^C NMR **(2)** (CDCl_3_, 125 MHz) δ 38.6 (CH_2_, C-1),26.9 (CH_2_, C-2), 79.0 (CHOH, C-3), 38.8 (C, C-4), 55.2 (CH, C-5), 18.4 (CH_2_, C-6), 32.7 (CH_2_, C-7), 39.8 (C, C-8), 47.6 (CH, C-9), 36.9 (C, C-10), 23.5 (CH_2_, C-11), 121.7 (HC=, C-12), 145.2 (C=, C-13), 41.7 (C, C-14), 27.2 (CH_2_, C-15), 26.2 (CH_2_, C-16), 32.5 (C, C-17), 47.2 (CH, C-18), 46.8 (CH_2_, C-19), 31.1 (C, C-20), 34.8 (CH_2_, C-21), 37.2 (CH_2_, C-22), 28.4 (CH_3_, C-23), 15.5 (CH_3_, C-24), 15.6 (CH_3_, C-25), 16.8 (CH_3_, C-26), 26.0 (CH_3_, C-27), 28.1 (CH_3_, C-28), 33.3 (CH_3_, C-29), 23.7 (CH_3_, C-30); EIMS **(1)** *m/z* 426 [M]^+^ (3), 411 (1), 218 (100), 203 (51), 189 (15); EIMS **(2)** *m/z* 426 [M]^+^ (8), 411 (2), 218 (100), 203 (22), 189 (19).

**Fraction PPM-2:** Isolated as an 2:1 mixture of brein **(3)** and maniladiol **(4)**; ^1^H NMR **(3)** (500 MHz, CDCl_3_)  3.23 (dd, *J*=10.9, 5.2 Hz), 4.20 (dd, *J*=11.6, 4.9 Hz, H-16), 5.20 ppm (*J* = 3.6 Hz, H-12); ^1^H NMR **(4)** (500 MHz, CDCl_3_)  3.23 (dd, *J*=10.9, 5.2 Hz, H-3), 4.22 (dd, *J*=11.3, 5.2 Hz, H-16), 5.26 ppm (*J* = 3.5 Hz, H-12); ^13^C NMR **(3)** (125 MHz, CDCl_3_) δ 38.8 (CH_2_, C-1), 27.2 (CH_2_, C-2), 79.0 (CHOH, C-3), 38.8 (C, C-4), 55.2 (CH, C-5), 18.3 (CH_2_, C-6), 32.9 (CH_2_, C-7), 40.1 (C, C-8), 47.00 (CH, C-9), 36.8 (C, C-10), 23.4 (CH_2_, C-11), 125.1 (HC=, C-12), 138.0 (C=, C-13), 44.1 (C, C-14), 36.0 (CH_2_, C-15), 67.0 (CH, C-16), 38.5 (C, C-17), 60.7 (CHOH, C-18), 39.5 (CH, C-19), 39.5 (CH, C-20), 30.5 (CH_2_, C-21), 35.2 (CH_2_, C-22), 28.1 (CH_3_, C-23), 15.6 (CH_3_, C-24), 15.7 (CH_3_, C-25), 16.9 (CH_3_, C-26), 24.5 (CH_3_, C-27), 21.3 (CH_3_, C-28), 17.6 (CH_3_, C-29), 21.9 (CH_3_, C-3). ^13^C NMR **(4)** (125 MHz, CDCl_3_) δ 38.6 (CH_2_, C-1), 27.2 (CH_2_, C-2), 79.0 (CHOH, C-3), 38.8 (C, C-4), 55.2 (C, C-5), 18.3 (CH_2_, C-6), 32.7 (CH_2_, C-7), 39.9 (C, C-8), 46.8 (CH, C-9), 37.3 (C, C-10), 23.5 (CH_2_, C-11), 122.3 (HC=, C-12), 143.5 (C=, C-13), 43.8 (C, C-14), 35.60 (CH_2_, C-15), 66.0 (CHOH, C-16), 36.9 (C, C-17), 49.1 (CH, C-18), 46.6 (CH_2_, C-19), 30.9 (C, C-20), 34.2 (CH_2_, C-21), 30.6 (CH_2_, C-22), 28.1 (CH_3_, C-23), 15.6 (CH_3_, C-24), 15.5 (CH_3_, C-25), 16.8 (CH_3_, C-26), 27.1 (CH_3_, C-27), 21.5 (CH_3_, C-28), 33.2 (CH_3_, C-29), 24.0 (CH_3_, C-30). EIMS **(3)** *m/z* 442 [M]^+^ (6), 424 (6), 234 (100), 219 (38), 191 (34), 110 (52); EIMS **(4)** *m/z* 442 [M]^+^ (4), 424 (2), 234 (100), 219 (26), 190 (16), 110 (10).

**Fraction PPM-3:** Isolated as an 2:1 mixture of 3α-hydroxytirucalla-7,24-diene-21-oic acid **(5)** and 3α-hydroxytirucalla-8,24-diene-21-oic acid **(6)**; ^1^H NMR **(3)** (500 MHz, acetone-D6) δ 0.79 (s, 3 H), 0.90 (s, 3 H), 0.92 (s, 3 H), 0.93 (s, 3 H), 1.00 (s, 3 H), 1.01(s, 3 H), 1.66 (s, 3 H), 3.39 (*t*, *J*=2,56 Hz), 5,12 (*tt*, *J*=7.17, 1.37 Hz, 1 H), 5,28 (m); ^1^H NMR **(6)** (500 MHz, Acetone-D6) δ 0.85 (s, 3 H), 0.87 (s, 3 H), 0.91 (s, 3 H), 0.95 (s, 3 H), 0.98 (s, 3 H), 1.58 (s, 3 H), 1.66 ppm (s, 3 H), 2.81 (brs), 3.36 (t, *J*=2,75), 5.12 ppm (tt, *J*=7.170, 1.373 Hz, 1 H); ^13^C NMR **(5)** (125 MHz, Acetone-D6) δ 32.2 (CH_2_, C-1), 26.6 (CH_2_, C-2), 75.9 (CHOH, C-3), 38.2 (C, C-4), 45.3 (CH, C-5), 24.8 (CH_2_, C-6), 119.3 (HC=, C-7), 146.8 (C=, C-8), 49.5 (CH, C-9), 35.6 (C, C-10), 27.8 (CH_2_, C-11), 34.4 (CH_2_, C-12), 44.2 (C, C-13), 51.9 (C, C-14), 31.4 (CH_2_, C-15), 17.8 (CH_2_, C-16), 50.6 (CH, C-17), 22.3 (CH_3_, C-18), 13.6 (CH_3_, C-19), 48.3 (CH, C-20), 177.3 (COOH, C-21), 33.4 (CH_2_, C-22), 25.9 (CH_2_, C-23), 124.9 (HC=, C-24), 132.3 (C=, C-25), 18.2 (CH_3_, C-26), 26.8 (CH_3_, C-27), 28.6 (CH_3_, C-28), 22.2 (CH_3_, C-29), 27.9 (CH_3_, C-30); ^13^C NMR **(6)** (125 MHz, Acetone-D6) δ 30.6 (CH_2_, C-1), 27.0 (CH_2_, C-2), 75.5 (CHOH, C-3), 38.5 (C, C-4), 45.5 (CH, C5), 19.6 (CH_2_, C-6), 28.2 (CH_2_, C-7), 133.8 (C=, C-8), 135.8 (C=, C-9), 38.1 (C, C-10), 27.6 (CH_2_, C-11), 29.7 (CH_2_, C-12), 44.8 (C, C-13), 50.5 (C, C-14), 30.2 (CH_2_, C-15), 22.1 (CH_2_, C-16), 47.8 (CH, C-17), 16.2 (CH_3_, C-18), 20.5 (CH_3_, C-19), 48.5 (CH, C-20), 177.4 (COOH, C-21), 33.5 (CH_2_, C-22), 27.0 (CH_2_, C-23), 124.9 (CH, C-24), 132.3 (C, C-25), 17.8 (CH_3_, C-26), 25.9 (CH_3_, C-27), 28.9 (CH_3_, C-28), 22.8 (CH_3_, C-29), 24.9 (CH_3_, C-30).

**Oxidation of PPM-1:** Obtained as an XX:X mixture of α-amyrone **(1a)** and β-amyrone **(2a);** ^1^H NMR **(1a)** (CDCl_3_, 500 MHz): δ 2.39 (m, H-2), 2.55 (m, H-2), 5.16 (t, *J*=3.7 Hz, H-12); ^1^H NMR **(2a)** (CDCl_3_, 500 MHz): δ 2.39 (m, H-2), 2.55 (m, H-2), 5.21 (t, *J*=3.7 Hz, H-12); ^13^C NMR **(1a)** (125 MHz, CDCl_3_) δ 39.5 (CH_2_, C-1), 34.2 (CH_2_, C-2), 217.8 (C=O, C-3), 47.4 (C, C-4), 55.3 (CH, C-5), 19.6 (CH_2_, C-6), 32.5 (CH_2_, C-7), 40.0 (C, C-8), 46.9 (CH, C-9), 36.6 (C, C-10), 23.5 (CH_2_, C-11), 124.2 (HC=, C-12), 139.7 (C=, C-13), 42.2 (C, C-14), 28.1 (CH_2_, C-15), 26.6 (CH_2_, C-16), 33.8 (C, C-17), 59.1 (CH, C-18), 39.7 (CH, C-19), 39.6 (CH, C-20), 31.2 (CH_2_, C-21), 41.5 (CH_2_, C-22), 26.6 (CH_3_, C-23), 21.4 (CH_3_, C-24), 15.4 (CH_3_, C-25), 16.8 (CH_3_, C-26), 23.2 (CH_3_, C-27), 28.8 (CH_3_, C-28), 17.5 (CH_3_, C-29), 21.5 (CH_3_, C-30). MS: *m/z* 424 [M]^+^ (10); 218 (100); 203 (27); 189 (18); ^13^C NMR **(2a)** (125 MHz, CDCl_3_) δ 39.3 (CH_2_, C-1), 34.2 (CH_2_, C-2), 217.7 (C=O, C-3), 47.4 (C, C-4), 55.3 (CH, C-5), 19.6 (CH_2_, C-6), 32.2 (CH_2_, C-7), 39.8 (C, C-8), 47.3 (CH, C-9), 36.7 (C, C-10), 23.6 (CH_2_, C-11), 121.5 (HC=, C-12), 145.2 (C=, C-13), 41.9 (C, C-14), 26.1 (CH_2_, C-15), 26.9 (CH_2_, C-16), 32.5 (C, C-17), 46.9 (CH, C-18), 46.8 (CH_2_, C-19), 31.1 (C, C-20), 34.7 (CH_2_, C-21), 37.1 (CH_2_, C-22), 26.5 (CH_3_, C-23), 15.2 (CH_3_, C-24), 15.4 (CH_3_, C-25), 16.7 (CH_3_, C-26), 25.9 (CH_3_, C-27), 28.4 (CH_3_, C-28), 33.3 (CH_3_, C-29), 23.7 (CH_3_, C-30). MS: *m/z* 424 [M]^+^ (9); 218 (100); 203 (52); 189 (19).

**COMPOUNDS**

| Table 1: Cell viability of J774 cells treated with 5, 10, and 20 μg/mL of isolated triterpenes for 24, 48 and 72 hours. | | | | | | | | | |
| --- | --- | --- | --- | --- | --- | --- | --- | --- | --- |
|  | 24 hours | | | 48 hours | | | 72 hours | | |
| Concentration (µg/mL) | 20  mean±SE | 10  mean±SE | 5  mean±SE | 20  mean±SE | 10  mean±SE  mean±SE | 5 | 20  mean±SE | 10  mean±SE | 5  mean±SE |
| αβ-amyrin | 57.3±1.9 | 86.4±0.8 | 105.8±2.3 | 36.4±3.1 | 88,5±2.8 | 110.0±2.5 | 37.1±3.5 | 88.7±1.2 | 97.2±1.8 |
| αβ-amyrin acetylated | 87.1±0.5 | 152.9±0.7 | 124.4±1.9 | 32.1±2.4 | 99.5±3.6 | 125.0±1.7 | 47.7±1.9 | 98.0±2.9 | 103.5±2.7 |
| αβ-amyrone | 119.4±0.5 | 153.8±1.8 | 144.0±0.78 | 81.9±2.9 | 126.7±0.7 | 133.5±1.5 | 65.3±1.7 | 102.0±0.7 | 105.2±3.5 |
| brein/maniladiol | 40.6±1.1 | 82.4±1.9 | 97.2±3.5 | 13.3±2.0 | 47.6±2.9 | 90.0±1.2 | 5.4±0.6 | 23.8±3.1 | 80.2±1.6 |
| Indomethacin | 100.1±3.2 | 123.6±2.9 | 117.5±3.7 | 50.5±2.8 | 100.8±1.3 | 105.9±0.8 | 57.9±1.1 | 102.3±1.7 | 103.8±2.9 |
| Doxorubicin | 26.0±0.4 | 27.3±1.2 | 25.7±2.6 | 9.3±1.4 | 10.0±0.1 | 10.1±0.2 | 3.8±0.7 | 3.9±0.07 | 3.9±0.1 |
| DMSO | 70.8±0.7 | 108.6±0.6 | 113.3±0.6 | 38.9±0.2 | 98.9±2.7 | 109.5±2.9 | 33.4±2.3 | 98.6±1.1 | 99.0±1.3 |
| Medium | 106.0616±4.0 | 100.01±9.7 | 101.30±1.5 | 106.7±1.8 | 101.9±11.0 | 103.4±2.1 | 99.99±4.8 | 98.99±3.4 | 95.4±4.2 |

**Notes:** Data are presented as % mean ± standard error (n=3). **Abbreviation:** SE, standard error; DMSO, dimethyl sulfoxide.

References

1. Corey EJ and Suggs, JW: Pyridinium chlorochromate. An efficient reagent for oxidation of primary and secondary alcohols to carbonyl compounds. *Tetrahedron Letters* 1975, 16 (31): 2647-2650.

2. Soldi C, Pizzolatti MG, Luiz AP, Marcon R, Meotti FC, Mioto LA, Santos ARS: Synthetic derivatives of the alpha- and beta-amyrin triterpenes and their antinociceptive properties. *Bioorganic & Medicinal Chemistry* 2008, 16 (6): 3377-3386.

3. Bruni S, Guglielmi V. [Identification of archaeological triterpenic resins by the non-separative techniques FTIR and 13C NMR: the case of Pistacia resin (mastic) in comparison with frankincense.](http://www.ncbi.nlm.nih.gov/pubmed/24291439) Spectrochim Acta A Mol Biomol Spectrosc. 2014;121:613-22.
